# Prostate cancer polygenic risk score and prediction of lethal prostate cancer

**DOI:** 10.1038/s41698-022-00266-8

**Published:** 2022-04-08

**Authors:** Robert J. Klein, Emily Vertosick, Dan Sjoberg, David Ulmert, Ann-Charlotte Rönn, Christel Häggström, Elin Thysell, Göran Hallmans, Anders Dahlin, Pär Stattin, Olle Melander, Andrew Vickers, Hans Lilja

**Affiliations:** 1 Department of Genetics and Genomic Sciences and Icahn Genomics Institute, Icahn School of Medicine at Mount Sinai, New York, NY 10029 USA; 2 Department of Epidemiology and Biostatistics, Memorial Sloan Kettering Cancer Center, New York, NY USA; 3 Department of Molecular and Medical Pharmacology, University of California Los Angeles, Los Angeles, CA USA; 4 Ahmanson Translational Imaging Division, David Geffen UCLA School of Medicine, Los Angeles, CA USA; 5 Jonsson Comprehensive Cancer Center, David Geffen UCLA School of Medicine, Los Angeles, CA USA; 6 Eli and Edythe Broad Center of Regenerative Medicine and Stem Cell Research, University of California Los Angeles, Los Angeles, CA USA; 7 Institute of Urologic Oncology, University of California Los Angeles, Los Angeles, CA USA; 8 Clinical Research Center, Karolinska University Hospital, Huddinge, Sweden; 9 Department of Biobank Research, Umeå University, Umeå, Sweden; 10 Department of Surgical Sciences, Uppsala University, Uppsala, Sweden; 11 Department of Medical Biosciences, Umeå University, Umeå, Sweden; 12 Department of Public Health and Clinical Medicine, Umeå University, Umeå, Sweden; 13 Department of Clinical Sciences, Lund University, Malmö, Sweden; 14 Department of Surgical Sciences, Uppsala University Hospital, Uppsala, Sweden; 15 Department of Emergency and Internal Medicine, Skåne University Hospital, Malmö, Sweden; 16 Departments of Laboratory Medicine, Surgery, and Medicine, Memorial Sloan Kettering Cancer Center, New York, NY USA; 17 Department of Translational Medicine, Lund University, Malmö, Sweden

**Keywords:** Prognostic markers, Prostate cancer, Disease genetics

## Abstract

Polygenic risk scores (PRS) for prostate cancer incidence have been proposed to optimize prostate cancer screening. Prediction of lethal prostate cancer is key to any stratified screening program to avoid excessive overdiagnosis. Herein, PRS for incident prostate cancer was evaluated in two population-based cohorts of unscreened middle-aged men linked to cancer and death registries: the Västerbotten Intervention Project (VIP) and the Malmö Diet and Cancer study (MDC). SNP genotypes were measured by genome-wide SNP genotyping by array followed by imputation or genotyping of selected SNPs using mass spectrometry. The ability of PRS to predict lethal prostate cancer was compared to PSA and a commercialized pre-specified model based on four kallikrein markers. The PRS was associated with incident prostate cancer, replicating previously reported relative risks, and was also associated with prostate cancer death. However, unlike PSA, the PRS did not show stronger association with lethal disease: the hazard ratio for prostate cancer incidence vs. prostate cancer metastasis and death was 1.69 vs. 1.65 in VIP and 1.25 vs. 1.25 in MDC. PSA was a much stronger predictor of prostate cancer metastasis or death with an area-under-the-curve of 0.78 versus 0.63 for the PRS. Importantly, addition of PRS to PSA did not contribute additional risk stratification for lethal prostate cancer. We have shown that a PRS that predicts prostate cancer incidence does not have utility above and beyond that of PSA measured at baseline when applied to the clinically relevant endpoint of prostate cancer death. These findings have implications for public health policies for delivery of prostate cancer screening. Focusing polygenic risk scores on clinically significant endpoints such as prostate cancer metastasis or death would likely improve clinical utility.

## Introduction

Various studies have identified hundreds of common genetic polymorphisms associated with the risk of developing prostate cancer. While each risk variant individually only confers a modest increase in a man’s risk of being diagnosed with prostate cancer, the combined effect can be substantial. By weighting and summing these polymorphisms in a polygenic risk score (PRS), it is possible to identify a subset of men at higher risk of developing prostate cancer. For instance, one study showed that men at the top 1% of the PRS had a 5.7-fold increased risk compared to the population average^1^. Similar results have been found using other prostate cancer PRS^2–5^.

Prostate cancer is generally a very slow-growing disease. The high prevalence of cancer in the autopsy prostate^6^—over 50% in older men—demonstrates that the vast majority of men who develop prostate cancer never experience symptoms before dying of another cause. The introduction of screening using prostate-specific antigen (PSA) led to widespread overdiagnosis, defined here as detection of cancer that would never have caused morbidity or mortality during the course of a patient’s natural life. It has been estimated that there were 1.3 million more US prostate cancer diagnoses than expected in the first 20 years of clinical use^7^, with about 1 million men overtreated, that is, undergoing curative therapy of an overdiagnosed cancer.

The use of incident prostate cancer as an endpoint for PRS studies will include overdiagnosed cancers. It therefore remains an open question whether PRS based on such endpoints will reduce overdiagnosis. To aid decisions about early detection of clinically significant disease, we need to focus screening efforts on men at high risk of lethal disease using endpoints of distant metastasis or death from prostate cancer. We have studied molecular predictors of prostate cancer mortality in blood collected at baseline in two large, well-annotated, and carefully ascertained population-based epidemiologic cohort studies of 55,300 Swedish men who were not subject to prostate cancer screening and were followed for 20 years^8,9^. Combined, there were 4001 incident cases of prostate cancer resulting in 679 prostate cancer deaths or distant metastasis in these cohorts. Here, we apply a PRS^1^ to these cohorts to determine whether it is predictive of prostate cancer death either alone or above and beyond blood markers collected many years before prostate cancer was diagnosed^8,9^: PSA or the 4Kscore, a commercially available pre-specified model based on four kallikrein markers used to inform biopsy decision-making in men with elevated PSA^10^.

## Results

### Assay design for PRS

We took two distinct strategies to measure the SNPs from the PRS. In the Malmö Diet and Cancer study (MDC), we matched the SNPs reported in the PRS^1^ to GWAS data previously imputed to SNPs in the 1000 Genomes reference panel^11^ and were able to match 145 out of 146 SNPs. In the Västerbotten Intervention Project (VIP) cohort, we aimed to genotype all 145 SNPs using a mass spectrometry-based approach. For SNPs for which we could not design a well-performing assay, we attempted to genotype proxy SNPs in strong linkage disequilibrium (*r*^2^ > 0.8). After two rounds of design and testing, followed by genotyping on the full nested case-control VIP cohort, we obtained high-quality measurements for 125 of the 146 SNPs (or their proxies). For consistency, our primary analyses focus on the risk score using this set of 125 SNPs in both the VIP and MDC cohorts (“Modified” score).

### Association of risk score with prostate cancer incidence and outcome

Details on the selection of men from these cohorts and their characteristics are presented (Tables 1 and 2; Supplementary Figs. 1 and 2). Men in the MDC ranged from age 48–73 although approximately half were in their 60 s at the time of blood draw. For the VIP cohort, 5%, 17%, and 78% in the cohort age within one year of 40, 50, and 60, respectively. Median follow-up among controls was 20 years (quartiles 17, 22) in the MDC cohort and 20 years (quartiles 17, 23) in the VIP cohort.

**Table 1:** Patient characteristics^a^ at baseline by case status^b^ in patients with available polygenic risk scores in the MDC and VIP case-control cohorts.

	MDC		VIP	
	Case (*N* = 240)^a^	Control (*N* = 751)^a^	Case (*N* = 235)^a^	Control (*N* = 765)^a^
Age at blood draw	65 (61, 70)	64 (60, 68)	60 (59, 60)	60 (59, 60)
Total PSA	3.82 (2.03, 7.25)	1.20 (0.68, 2.38)	2.61 (1.56, 5.32)	1.07 (0.67, 1.93)
Free PSA	0.71 (0.42, 1.18)	0.37 (0.24, 0.66)	0.55 (0.35, 0.85)	0.34 (0.23, 0.54)
Intact PSA	0.41 (0.23, 0.65)	0.20 (0.12, 0.36)	0.37 (0.22, 0.58)	0.23 (0.15, 0.35)
hK2	0.055 (0.037, 0.086)	0.032 (0.022, 0.050)	0.051 (0.033, 0.085)	0.033 (0.022, 0.048)
Modified PRACTICAL risk	10.85 (10.42, 11.22)	10.53 (10.10, 11.01)	10.40 (10.01, 10.84)	10.06 (9.65, 10.53)

^a^Data are presented as median (quartiles).

^b^Case status is prostate cancer death in the MDC cohort and distant metastasis or prostate cancer death in the VIP cohort.

**Table 2:** Cancer characteristics at diagnosis for incident cases in the full population data for the MDC cohort and the cancer cases from the full nested case-control VIP cohort.

Characteristic^a^	MDC, *N* = 1576	VIP, *N* = 2326
PSA at diagnosis	10 (6, 23)	10 (6, 20)
Unknown	299	825
Biopsy grade		
6	618 (43%)	814 (53%)
7 (3 + 4)	248 (17%)	0 (0%)
7 (4 + 3)	118 (8.2%)	0 (0%)
7	10 (0.7%)	469 (31%)
8	114 (7.9%)	136 (8.9%)
9	155 (11%)	98 (6.4%)
10	13 (0.9%)	8 (0.5%)
WHO Grade 1	84 (5.8%)	0 (0%)
WHO Grade 2	51 (3.5%)	0 (0%)
WHO Grade 3	27 (1.9%)	0 (0%)
Unknown	138	801
Clinical T stage		
T0	21 (1.4%)	3 (0.2%)
T1	118 (8.1%)	0 (0%)
T1A	37 (2.6%)	45 (3.0%)
T1B	36 (2.5%)	17 (1.1%)
T1C	423 (29%)	725 (49%)
T2	454 (31%)	507 (34%)
T3	320 (22%)	178 (12%)
T4	41 (2.8%)	18 (1.2%)
Unknown	126	833
Clinical N stage		
N0	310 (21%)	275 (18%)
N1	34 (2.3%)	25 (1.6%)
Nx	1106 (76%)	1227 (80%)
Unknown	126	799
Clinical M stage		
M0	763 (53%)	694 (45%)
M1	125 (8.6%)	125 (8.2%)
Mx	562 (39%)	708 (46%)
Unknown	126	799
Metastatic at diagnosis		
No	115 (7.3%)	695 (30%)
Yes	95 (6.0%)	125 (5.4%)
Unknown	1266 (87%)	1506 (65%)
Distant metastasis	–	296
Unknown	1576	0
Prostate cancer death	288	168

When we tested the modified risk score in our cohorts, we found a significant association with incident diagnosis of any grade prostate cancer with similar hazard ratios to those previously reported^1^ (*p* < 0.001; Tables 3 and 4). Similar results were found when we computed the risk score using all 145/146 variants for which we had 1000 Genomes imputation data in the MDC (Tables 3 and 4). We also found that the PRS was significantly associated with the endpoint of distant metastasis or death from prostate cancer (*p* < 0.001; Table 5). Importantly, however, the PRS could not differentially distinguish higher risk disease (Table 5) with similar estimates irrespective of endpoint; most notably, the hazard ratio for prostate cancer incidence (1.69 in VIP and 1.25 in MDC) was nearly identical to that for prostate cancer metastasis and death (1.65 in VIP and 1.25 in MDC).

**Table 3:** Association between high-risk score^a^ vs average risk score^b^ and the outcome of incident diagnosis of any grade prostate cancer.

Cohort	Risk score	Event N	Average score *N*	High score *N*	OR (95% CI)	*p* value
MDC	PRACTICAL risk (145 SNPs)	524	1898	69	3.35 (2.07, 5.46)	<0.001
MDC	Modified PRACTICAL risk	554	1925	77	2.97 (1.88, 4.71)	<0.001
VIP	Modified PRACTICAL risk	669	2525	82	5.99 (3.79, 9.66)	<0.001
Both	Modified PRACTICAL risk	1223	4450	159	4.25 (3.09, 5.90)	<0.001

^a^High-risk score is defined as ≥99th centile

^b^Average risk score is defined as 25–75th centile

**Table 4:** Association between high-risk score^a^ vs average risk score^b^ and the outcome of incident diagnosis of any grade prostate cancer.

Cohort	Risk score	Event N	Average score N	High score N	OR (95% CI)	*p* value
MDC	PRACTICAL risk (145 SNPs)	723	1898	527	2.35 (1.92, 2.87)	<0.001
MDC	Modified PRACTICAL risk	733	1925	503	2.12 (1.73, 2.59)	<0.001
VIP	Modified PRACTICAL risk	893	2525	673	2.19 (1.83, 2.61)	<0.001
Both	Modified PRACTICAL risk	1626	4450	1176	2.15 (1.88, 2.46)	<0.001

^a^High-risk score is defined as 90–99th centile

^b^Average risk score is defined as 25–75th centile

**Table 5:** Association between polygenic risk scores and four nested outcomes in the VIP and MDC cohorts separately.

	VIP cohort		MDC cohort	
	Event N	Modified PRACTICAL risk^a^	Event *N*	Modified PRACTICAL risk^a^
Prostate cancer	2412	1.69 (1.60, 1.79)	1576	1.25 (1.11, 1.41)
Intermediate and high-risk prostate cancer	1167	1.74 (1.64, 1.84)	1120	1.26 (1.11, 1.43)
High-risk prostate cancer	552	1.68 (1.54, 1.83)	625	1.18 (1.02, 1.36)
Prostate cancer metastasis or death	308	1.65 (1.48, 1.84)	371	1.25 (1.10, 1.41)

^a^Hazard ratio per 1 standard deviation increase (95% Confidence Interval)

### Comparison with PSA and 4Kscore

We then went on to investigate the incremental value of this PRS compared to midlife levels of PSA or the 4Kscore^8,9^. The risk score remained significantly associated with prostate cancer distant metastasis or death after adjusting for either midlife PSA or the 4Kscore (Table 6). However, it did not improve discrimination over total PSA in all men (change in AUC −0.009) or in men with elevated PSA who might consider biopsy (change in AUC 0.006). The risk score did not improve the discrimination of the 4Kscore in men with higher PSA (Table 7). Importantly, the single PSA measurement predicts risk of future distant metastasis or death from prostate cancer far better than the PRS, with an AUC of 0.78 compared to 0.63.

**Table 6:** Association between modified PRACTICAL risk score and prostate cancer metastasis or death in both the VIP and MDC cohorts on univariate analysis and adjusted for total PSA and 4kscore among PSA subgroups.

			Modified^a^ PRACTICAL risk only			Modified PRACTICAL risk + total PSA			Modified PRACTICAL risk + 4Kscore		
PSA range	Total *N*	Case *N*	OR^b^	95% CI	*p* value	OR^b^	95% CI	*p* value	OR^b^	95% CI	*p* value
PSA ≥ 0	1991	475	1.07	1.05, 1.08	<0.001	1.04	1.03, 1.06	<0.001	1.05	1.03, 1.06	<0.001
PSA ≤ 1	722	46	1.05	1.01, 1.10	0.026	1.05	1.00, 1.09	0.034	1.05	1.00, 1.09	0.029
PSA ≥ 1	1278	433	1.05	1.03, 1.07	<0.001	1.04	1.02, 1.06	<0.001	1.03	1.02, 1.05	<0.001
PSA ≥ 2	754	336	1.04	1.02, 1.06	<0.001	1.03	1.01, 1.05	0.009	1.02	1.00, 1.04	0.047
PSA ≥ 3	506	249	1.05	1.02, 1.07	<0.001	1.04	1.01, 1.07	0.004	1.03	1.00, 1.06	0.023
PSA ≥ 4	363	202	1.05	1.02, 1.08	0.003	1.04	1.01, 1.08	0.008	1.03	1.00, 1.07	0.045

^a^125-SNP score.

^b^Odds ratios presented are per 0.1 unit increase in modified PRACTICAL risk.

**Table 7:** Discrimination for three risk markers measured at baseline and change in discrimination when adding Modified PRACTICAL risk to total PSA and the 4kKscore for the outcome of prostate cancer metastasis or death.

	Modified PRACTICAL risk	Total PSA	Modified PRACTICAL risk + Total PSA	Change in AUC from total PSA alone	4Kscore	Modified PRACTICAL risk + 4Kscore	Change in AUC from 4Kscore alone
All men	0.630	0.782	0.772	−0.009	0.758	0.761	0.003
PSA ≤ 1.0^a^	0.603	0.595	0.620	0.025	–	–	–
PSA ≥ 3.0^b^	0.580	0.649	0.655	0.006	0.724	0.724	0.000

^a^The PSA ≤ 1.0 ng/ml cutpoint concerns the clinical decision about the frequency of screening.

^b^The PSA ≥ 3.0 ng/ml cutpoint concerns the clinical decision about biopsy.

### Sensitivity analyses

We assessed the possibility that excluding the modifications we made to the PRACTICAL score due to the genotyping constraints in the VIP cohort affected the performance of the score. We calculated the PRACTICAL risk score, using the 145 out of 146 SNPs that were available in the MDC cohort, and repeated our analyses. Results were consistent with the modified PRACTICAL risk score analysis using both cohorts, with evidence of an improvement in discrimination above and beyond that of total PSA only for the men with PSA ≤ 1 ng/ml at baseline, but no improvement in discrimination for other PSA subgroups above and beyond that of total PSA or the 4Kscore measured at baseline (Supplementary Table 1). As a second sensitivity analysis, we repeated all analyses including the 50 men with PSA > 25 ng/ml at baseline; results were consistent with our main analysis (data not shown).

As numerous other PRSs for incident prostate cancer have recently been proposed^3,4,12,13^, as a sensitivity analysis we asked if any of these scores would improve the AUC when added to either midlife PSA or the 4KScore. As with the primary PRS we tested, no improvement in was observed by adding in a PRS (Supplementary Table 2).

### Evaluation of PRS measured before blood-based biomarker measure

**Fig. 1:**
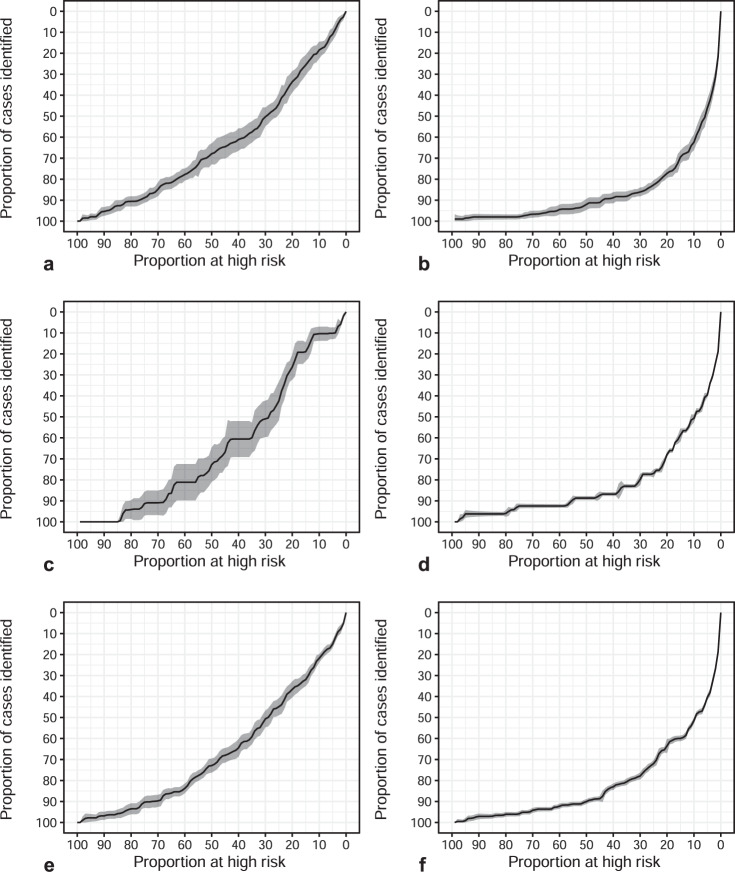
Lorenz curves for the modified PRACTICAL risk score (125 SNPs) and PSA in each of the MDC, VIP at age 50, and VIP at age 60 cohorts. **a** PRS in MDC. **b** PSA in MDC. **c** PRS in VIP age 50. **d** PSA in VIP age 50. **e** PRS in VIP age 60. **f** PSA in VIP age 60.

As genomic analyses become more common in the clinic, it is not unlikely that a man already had a baseline profile of common genetic variants measured prior to any screening. We therefore asked whether the PRS could allow pre-screening men to identify those who would not benefit from additional PSA testing. We asked what fraction of the cases who either had distant metastasis or death within 15 years (VIP cohort) or death from prostate cancer within 15 years (MDC cohort) would be found in various upper quantiles of the PRS. While we found that there was an excess of cases identified in the various quantiles of higher risk, it was not enough to be clinically meaningful (Fig. 1). For instance, in the MDC cohort, focusing on individuals in the top half of the PRS distribution would identify only 72% of the lethal prostate cancer cases. In contrast, the top half of the PSA distribution (PSA > 1 ng/ml) accounts for 92% of the lethal prostate cancer cases.

**Fig. 2:**
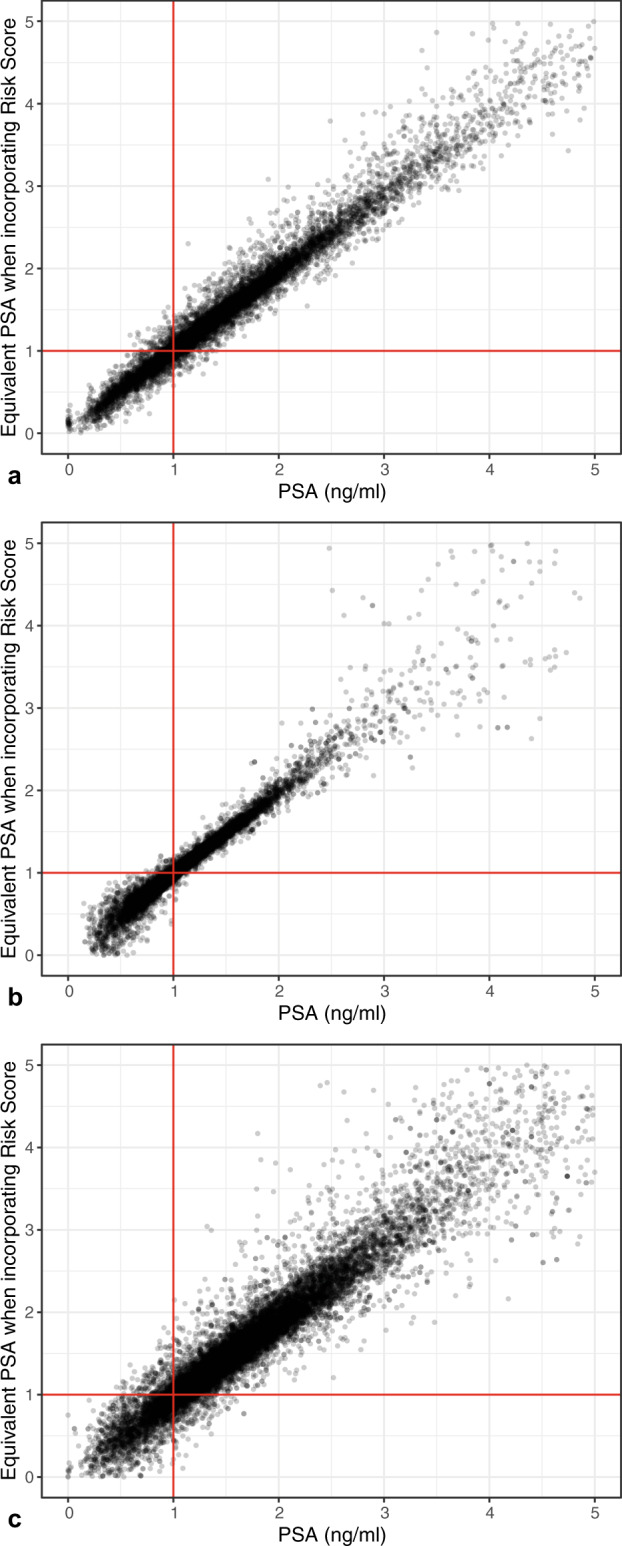
Reclassification by modified PRACTICAL Risk score (125 SNPs). Reclassification shown in **a** the MDC cohort, **b** men sampled at age 50 in the VIP cohort, and **c** men sampled at age 60 in the VIP cohort. The *x*-axis is total PSA, and the y-axis is the equivalent total PSA based on predicted probability after incorporating the PRACTICAL risk score, for the outcome of prostate cancer metastasis or death (VIP) or prostate cancer death (MDC) within 20 years.

We found a potentially promising increase in AUC of the PRS for men with PSA ≤ 1 ng/ml (change in AUC 0.025), a threshold that has been proposed to determine the frequency of screening or whether to cease screening^14,15^. However, in a reclassification analysis, among 16,236 men with a PSA ≤ 1 ng/ml, 977 were reclassified to a risk greater than that associated with PSA of ≤1 ng/ml, with a small absolute change in risk. Similarly, among the 34,534 men with a PSA > 1 ng/ml, 1465 were reclassified (Fig. 2), with the change in absolute risk being small. The overall reclassification rate was 5%.

## Discussion

We have shown that a PRS^1^ can not only predict the risk of incident diagnosis of any prostate cancer in independent cohorts but also predict which men may die from prostate cancer. Critically important however, the predictive value of this PRS is similar across increasingly aggressive phenotypes. There was no evidence that it provided additional clinical value to currently used biomarkers in aiding decision about biopsy or screening frequency.

Other PRS have been proposed for prostate cancer^2–5^ that were developed using similar statistical approaches and with the same endpoint of incident prostate cancer. The one PRS study that did consider mortality as an endpoint found that the PRS designed for incidence predicts mortality no better than incident cancer^4^. Unsurprisingly, in our sensitivity analysis, we found that none of these PRS compared favorably with using PSA testing alone. Thus, we consider it unlikely that improved PRS for incident prostate cancer will be sufficient to allow PRS to outperform PSA either singly or in combination.

We note recent proposals that prostate cancer screening strategies could be guided by germline genetic testing for prostate cancer risk. For instance, one group proposing a PRS explicitly recommend in the title of their paper that their marker should “guide screening for aggressive prostate cancer”^2^. Other groups have proposed^16^, based on mathematical models, that a “tailored screening program” based on polygenic risk, could “reduce overdiagnosis and improve the benefit-harm tradeoff”. Our data suggest that work to bring such proposals to fruition should focus on predictors of prostate cancer metastasis and death in the population, to avoid overdiagnosis of prostate cancer that is unlikely to reduce quality or length of life. Our findings might also help explain the disappointing results of the BARCODE1 study^17^, which investigated a strategy where men with a high PRS were sent straight to prostate biopsy. In a pilot study of 1434 men, this resulted in the detection of seven low-grade but no high-grade prostate cancers. We propose that PRSs that show stronger association with aggressive disease than indolent disease will have greater clinical utility.

While current prostate cancer risk SNPs are independent of disease aggressiveness, other yet to be identified SNPs may influence development of aggressive disease. It is interesting to note that these polygenic scores only consider common genetic variants in light of recent work showing that rare coding variants in DNA repair genes predispose to especially aggressive prostate cancer^18^. Polygenic models that are expanded to include such rare variants, as well as genetic factors influencing outcome independent of risk^11^ may better predict risk of lethal prostate cancer.

Our analyses are limited to individuals of European ancestry. As the scores tested were developed in this population, this does not detract from our primary findings. Nonetheless, research should examine whether variants associated with risk of overall prostate cancer in men of African ancestry are those also associated with the lethal phenotype. Despite needing to substitute or eliminate some SNPs for technical reasons in the analysis of the VIP cohort, we found in the MDC cohort that this modified 125-SNP score gave results that were strikingly similar to the full 145-SNP score, suggesting that this PRS is robust to slight changes in the SNPs used. As PRSs incorporate larger numbers of common variants with small effect sizes^19^, it is likely that genome-wide genotyping followed by imputation will be necessary to replicate the scores efficiently in external cohorts.

Our findings should not be generalized to impugn genetic risk scores in general. First, it is likely that a risk score designed for the endpoint of lethal prostate cancer would perform better. Second, our results are only pertinent to clinical utility of this score: the genetic loci, and target genes, implicated by GWAS retain their value for understanding prostate cancer etiology and providing potential targets in terms of chemoprevention or treatment.

The natural history of prostate cancer is that most men will die with rather than of disease. Screening for prostate cancer has led to widespread overtreatment and overdiagnosis. We have demonstrated that a PRS that predicts prostate cancer incidence does not have utility above and beyond that of PSA measured at baseline when applied to the clinically relevant endpoint of prostate cancer death. Therefore, using the PRS to determine which men should be subject to prostate cancer screening will not change the proportion subject to overdiagnosis and overtreatment. Subsequent research should focus on the clinically relevant endpoints of prostate cancer morbidity and mortality.

## Methods

### Data collection

Data from two large population-based studies were used for these analyses: the MDC and the VIP. These studies were approved by local institutional review boards (Research Ethics Board at Umeå University, number 2009-1436-31, for VIP and the Research Ethics Board at Lund University, numbers 617/2005 and LU 425-02, for MDC) and written informed consent was obtained from each participant in accordance with the principles of the Declaration of Helsinki. In the MDC study, blood samples were taken between 1991 and 1996 from 11,506 men born between 1923 and 1945 and living in Malmö, Sweden. Data on follow-up for prostate cancer diagnosis and death from prostate cancer were collected until December 31, 2014. A nested matched case-control set was selected from the full cohort, with men diagnosed with prostate cancer selected as cases, and controls matched based on age and time of blood draw. Men selected as cases or controls had kallikrein markers measured using their stored blood samples. Kallikrein marker measurements were imputed for those men not selected as cases or controls. Additional details on cohort selection and data collection have previously been published^8^.

The VIP is an ongoing population-based cohort study initiated in 1985 in which all residents of Västerbotten County, Sweden, are invited to receive a health examination at ages 40, 50, and 60 with blood drawn for cryopreservation. By February 2017, the study included data on 58,398 samples from 43,692 unique men. A nested matched case-control sample was also selected from the VIP cohort based on age and time of blood draw, with cases being defined as those diagnosed with prostate cancer. Kallikrein markers were measured from blood samples for those men selected as cases and controls. Kallikrein marker measurements were imputed for those men not selected as cases or controls. Details on cohort selection, case-control matching and data collection in this cohort have also been previously published^9,20^.

The original case-control sets were based on cases diagnosed with prostate cancer. As our endpoint of interest was prostate cancer metastasis or death, we re-selected a nested, matched case-control set from both the VIP and MDC cohorts separately. In these two case-control sets, cases were those men who had SNP information and kallikrein measurements available and had distant prostate cancer metastasis or died from prostate cancer. Three controls were identified for each case, with each control being event-free at the time of the matched case metastasis or death and within one year of the case’s age at blood draw.

### Laboratory methods

All laboratory analyses were conducted blind to outcome and case-control status. We measured total and free PSA with the dual-label DELFIA ProStatus assay (PerkinElmer, Turku, Finland), calibrated against the World Health Organization (WHO) 96/670 (PSA-WHO) and WHO 68/668 (free PSA-WHO) standards, in previously unthawed cryopreserved heparin anticoagulated blood plasma. Intact PSA and hK2 were measured using F(ab’)2 fragments of the monoclonal capture antibodies to reduce the frequency of nonspecific assay interference and carried out as previously reported^21,22^.

### Genetic methods

For the MDC, all prostate cancer cases were genotyped on a variant of the Illumina Omni2.5 platform, along with a subset of controls who were included in genotyping for other, non-cancer, studies on the MDC. After quality control, individual SNP profiles were phased with ShapeIT and imputed to the 1000 Genomes reference panel using IMPUTE2. SNPs of interest were then selected from the imputed data. For the MDC, SNP genotypes were considered on a continuous scale of 0–2 representing the expected allele dosage for the risk allele. We consider this subset of SNPs, with the same weights, to be representative of the original polygenic score. We used an automated process to match the SNPs in scores from the polygenic score catalog^23^ for our sensitivity analysis.

For VIP, we selected a proxy SNP on the basis of LD in the “EUR” (European ancestry) population in 1000 Genomes when an assay could not be designed or tested for the original SNP in the PRACTICAL PRS, or removed the SNP from the risk score if that proxy could not be designed or successfully tested and kept the same weights for proxies as those used in the PRS, recognizing that the actual estimate of the weight could be different for the proxy SNP in the original PRACTICAL dataset. The MassArray assays were then performed on all 6920 DNA samples from the nested case-control set of the VIP cohort. After accounting for SNPs for which assays could not be designed, replacing some of them with tags in strong linkage disequilibrium, and quality control of the resulting genotypes, we were able to obtain good quality genotypes for 125 of 138 SNPs attempted. VIP SNPs were coded as 0, 1, or 2 based on the observed count of risk alleles. We then used these SNPs to create a “Modified PRACTICAL” score, which we computed in both the MDC and VIP for consistency.

For VIP, when a genotype was missing we estimated the genotype value as the mean of the genotypes across the other individuals. The PRS was then computed as **Σ***β*_*i*_*x*_*i*_ where *β*_*i*_ is the log of the per-allele odds ratio for SNP *i* and *x*_*i*_ represents the expected number of risk alleles(0–2) for the SNP present in the individual.

### Statistical methods

We began by attempting to replicate the PRACTICAL risk^1^ scores as reported in the original papers. To replicate the results of the PRACTICAL paper, a case-control dataset matched on any grade prostate cancer was used, and patients in the 99th centile or higher and patients in the 90–99th centiles were compared to those patients in the 25–75th centiles. Centiles of risk were calculated among patients without cancer in the full population data. Our primary analysis contains 125/146 SNPs that were available in both the VIP and MDC. We also conducted sensitivity analysis with a second modified PRACTICAL risk score that contains all SNPs available in the MDC (145/146), including those not available in the VIP; this analysis was conducted in the MDC only.

We then aimed to determine whether any of the PRSs predicted incident diagnosis of any grade prostate cancer, intermediate risk prostate cancer (clinical stage T2 or Gleason grade 7 or PSA ≥ 10 ng/ml and ≤20 ng/ml), high risk prostate cancer (clinical stage ≥ T3 or clinical stage N1 or clinical stage M1 or Gleason grade ≥ 8 or PSA > 20 ng/ml), or prostate cancer metastasis or death. In the VIP cohort, patients may have had multiple samples taken, so we selected the first observation for each patient. The risk scores were imputed separately in the VIP and MDC cohorts for those patients who were missing each score. The scores were standardized within each cohort by dividing by standard deviation. Cox proportional hazards models were created for time to each of the four outcomes, with the hazard ratio representing a 1 standard deviation increase in PRS.

We also assessed whether any of the PRSs added predictiveness above and beyond that based on total PSA or the 4Kscore measured at baseline. The four kallikrein markers were combined as previously described into a pre-defined statistical risk prediction model that gives the risk of ISUP Grade Group ≥ 2 prostate cancer (Gleason score ≥ 7) at prostate biopsy. This model is the same as the commercially available 4Kscore™, a comprehensively evaluated statistical model that is widely used in clinical practice^10,24^ and was completed and locked-down before application to these cohorts. We assessed the association between the risk score and case status using univariate logistic regression in two case-control cohorts (VIP and MDC) with controls matched on prostate cancer metastasis or death. Two multivariable logistic regression models were then used to test this association adjusting for either total PSA or 4Kscore at baseline. These associations were tested in all men, and in subgroups defined by PSA-level at baseline. Discrimination was calculated as the AUC. Patients with a PSA ≥ 25 ng/ml at baseline (*N* = 28 in MDC and *N* = 22 in VIP) were included only in a sensitivity analysis.

To evaluate if the PRS would be useful to pre-screen individuals before PSA testing, we used Lorenz curve analysis. We focused on a binary outcome: distant metastasis or prostate cancer death within 15 years (VIP cohort) or death from prostate cancer within 15 years (MDC cohort). For men in the control group who did not have total PSA and/or PRACTICAL Risk Score measured, these values were imputed using multiple imputation. For the VIP cohort, graphs were created separately for age 50 and age 60 cohorts. There are two versions of the PRACTICAL Risk score available. One version includes 145/146 SNPs in the original PRS^1^, but includes SNPs that are not available in the VIP cohort, and is therefore only available in the MDC cohort. The second modified version includes 125/146 SNPs, and only includes SNPs available in both MDC and VIP cohorts. For comparison, the Lorenz curves for total PSA in each cohort are also provided. Lorenz curve estimates were calculated in each of the ten imputed datasets. The mean across the ten imputations was used as the central estimate and the standard errors were calculated using Rubin’s rules. All analyses were performed using R 3.6.0.

### Reclassification analysis

To assess the clinical utility of the PRS, a reclassification analysis was performed. The full MDC and VIP cohorts were used, including men with imputed marker measurements and risk scores. We used predictive mean matching to impute marker levels and PRSs for men who were included in the VIP and MDC cohorts but were not selected for marker measurement or genotyping. Imputation was performed separately for men aged 40, 50, and 60 in the VIP cohort. Marker values (total PSA, free PSA, intact PSA and hk2), modified PRACTICAL risk and modified Seibert hazard were imputed based on case status for the outcomes of incident any grade prostate cancer diagnosis (n = 213 with both markers and risk scores imputed, *n* = 60 with only markers imputed and *n* = 101 with only risk scores imputed in MDC; *n* = 942 with both markers and risk scores imputed, *n* = 49 with only markers imputed, *n* = 482 with only risk scores imputed in VIP), distant metastasis (*n* = 6 with both risk scores and markers imputed and 38 with only risk scores imputed in VIP) and prostate cancer death (n = 65 with both markers and risk scores imputed, *n* = 15 with only marker imputed and *n* = 27 with only risk scores imputed in MDC; *n* = 13 with both markers and risk scores imputed, *n* = 24 with risk scores only imputed in VIP). Statistical analyses were performed on the population level utilizing the measured and imputed values combined across ten imputations using Rubin’s rules.

These analyses were performed separately in each cohort, and separately by age in the VIP cohort. Two Cox proportional hazards models for the outcome of time to prostate cancer metastasis or death were created, one containing total PSA or the 4kscore, and the other containing total PSA or the 4kscore and the risk score. Restricted cubic splines were included for total PSA to account for non-linearity. The predicted probability of prostate cancer metastasis or death within 20 years was calculated from each model. We then calculated the number of men for whom a clinical decision to continue or not continue screening would change based on the addition of the risk score—that is, whether adding the risk score to total PSA would raise the risk in a patient with a PSA ≤ 1 ng/ml to a risk equivalent with a PSA > 1 ng/ml, or vice versa.

### Reporting summary

Further information on research design is available in the Nature Research Reporting Summary linked to this article.

## Supplementary information

Supplemental Figures and Table

REPORTING SUMMARY

## Data Availability

The datasets generated during and/or analyzed during the current study are not publicly available to maintain compliance with European data protection laws. Anonymized data are available after application to the MDCS Steering Committee (https://www.malmo-kohorter.lu.se/malmo-cohorts).

## Code availability

The custom code used to generate these specific anlyses are available from the authors upon request.
