# Supplementary material for: Prostate cancer polygenic risk score and prediction of lethal prostate cancer
Source: NPJ Precis Oncol. 2022 Apr 8;6:25. doi: 10.1038/s41698-022-00266-8 (PMC8993880; doi:10.1038/s41698-022-00266-8)
Supplement: Supplementary file 1 — Supplemental Figures and Table [file 41698_2022_266_MOESM1_ESM.pdf]

Supplementary Figure 1. Flowchart for the MDC Study.

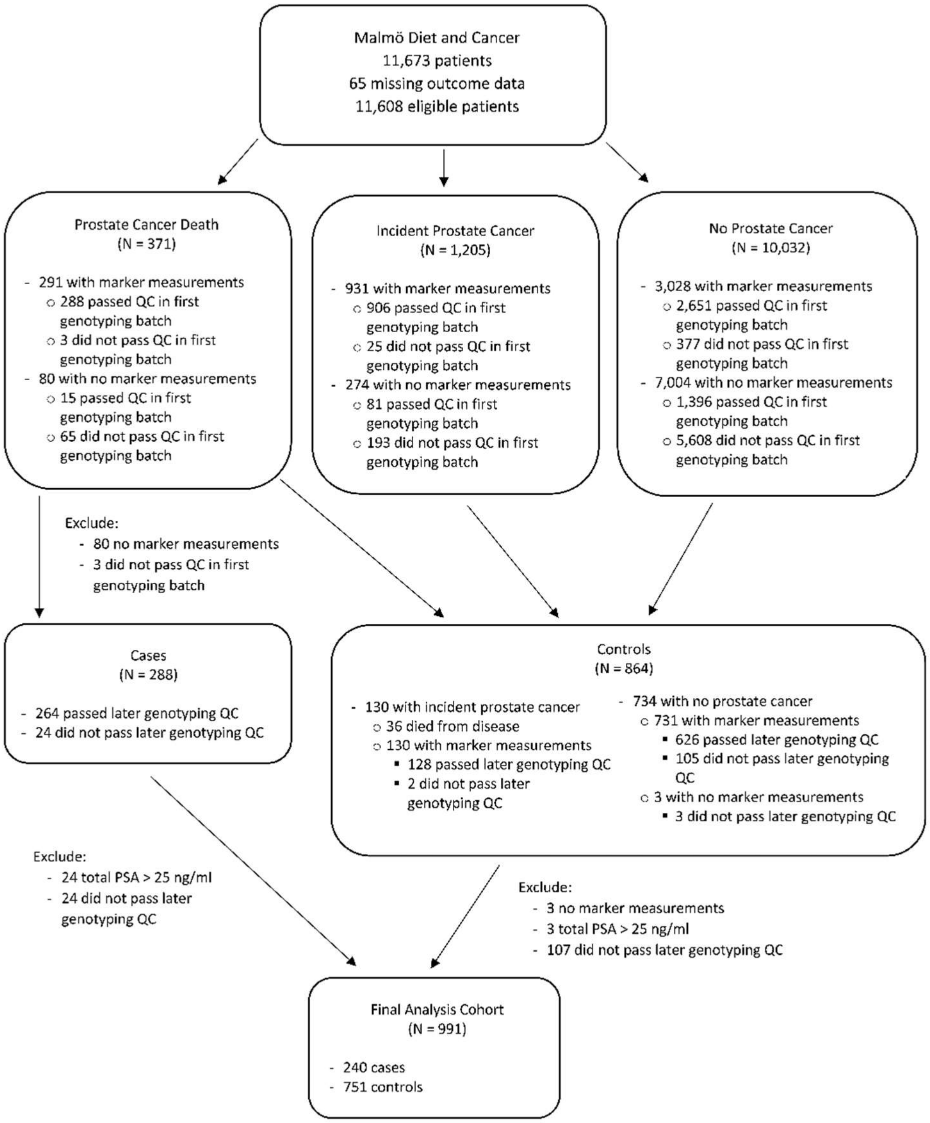

Supplementary Figure 2. Flowchart for the VIP cohort.

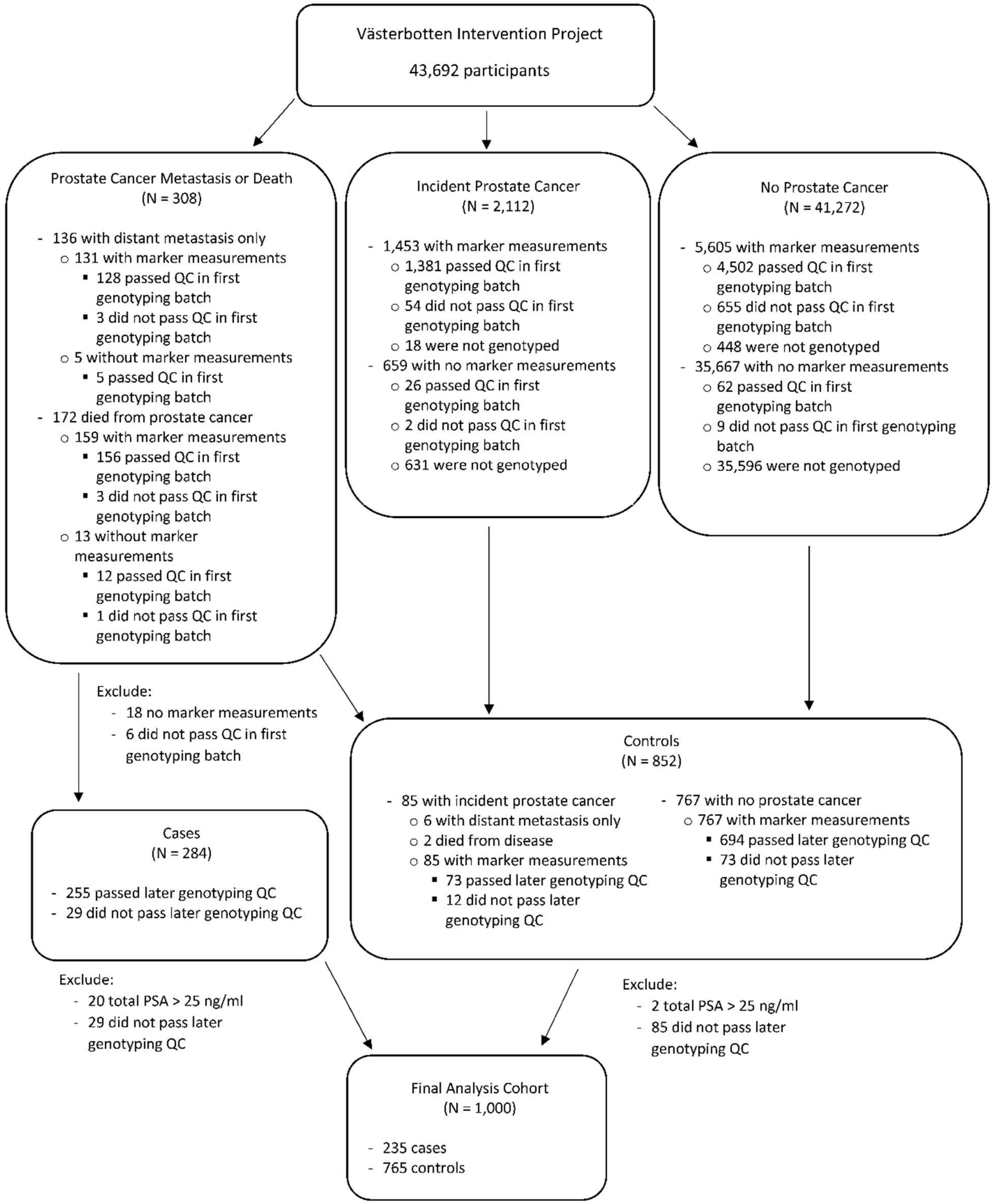

Supplementary Table 1. Discrimination for 145-SNP Practical risk score, total PSA and the 4kscore, and change in discrimination when adding this score to total PSA and the 4kscore for the outcome of prostate cancer death, in the MDC cohort only. Change in discrimination is presented only when MDC practical risk score is associated with outcome at p <= 0.1.

	Practical Risk Score (145-SNP)	Total PSA	Practical Risk Score (145-SNP) + Total PSA	Change in AUC from Total PSA Alone	4kscore	Practical Risk Score (145-SNP) + 4kscore	Change in AUC from 4kscore Alone
PSA ≥ 0	0.634	0.792	0.787	-0.005	0.769	0.780	0.011
PSA ≤ 1	0.675	0.671	0.713	0.043	---	---	---
PSA ≥ 1	0.596	0.721	0.716	-0.006	0.750	0.750	-0.001
PSA ≥ 2	0.567	0.644	0.645	---	0.717	0.716	---
PSA ≥ 3	0.568	0.650	0.652	0.002	0.713	0.720	---
PSA ≥ 4	0.561	0.576	0.613	---	0.695	0.704	---

Supplementary Table 2. AUCs for all selected polygenic risk scores for the outcome of death from prostate cancer in the MDC cohort, including all men regardless of PSA.

PRS	SNPs matched / SNPs in PRS	PRS Only	Total PSA	PRS + Total PSA	Change in AUC from Total PSA Alone	4Kscore	PRS + 4Kscore	Change in AUC from 4Kscore Alone
pgs000030	143/147	0.623	0.808	0.782	-0.025	0.787	0.794	0.007
pgs000044	66/66	0.593	0.808	0.782	-0.026	0.787	0.792	0.005
pgs000049	103/103	0.641	0.808	0.781	-0.026	0.787	0.795	0.008
pgs000067	46/54	0.530	0.808	0.796	-0.011	0.787	0.787	0.000
pgs000084	161/161	0.565	0.808	0.779	-0.029	0.787	0.779	-0.008
pgs000160	79/79	0.597	0.808	0.784	-0.024	0.787	0.786	-0.001
pgs000333	6605953/6606785	0.620	0.808	0.775	-0.032	0.787	0.783	-0.004
pgs000348	34/72	0.568	0.808	0.785	-0.022	0.787	0.782	-0.005
pgs000565	21/21	0.579	0.808	0.778	-0.030	0.787	0.785	-0.002
pgs000566	1111494/1111494	0.564	0.808	0.790	-0.018	0.787	0.785	-0.002
pgs000567	78/78	0.560	0.808	0.793	-0.015	0.787	0.794	0.007
pgs000568	1398/1401	0.591	0.808	0.782	-0.025	0.787	0.795	0.008
pgs000569	49/49	0.600	0.808	0.773	-0.035	0.787	0.783	-0.004
pgs000570	1111493/1111493	0.608	0.808	0.775	-0.033	0.787	0.782	-0.006
pgs000571	100/100	0.598	0.808	0.770	-0.038	0.787	0.779	-0.008
pgs000572	1808/1809	0.604	0.808	0.774	-0.034	0.787	0.788	0.001
pgs000573	31/31	0.608	0.808	0.771	-0.037	0.787	0.781	-0.006
pgs000574	1111493/1111493	0.575	0.808	0.781	-0.026	0.787	0.783	-0.004
pgs000575	47/47	0.618	0.808	0.774	-0.034	0.787	0.784	-0.004
pgs000576	904/905	0.601	0.808	0.774	-0.033	0.787	0.784	-0.003
pgs000577	117/117	0.616	0.808	0.784	-0.024	0.787	0.794	0.007
pgs000578	117/117	0.616	0.808	0.784	-0.024	0.787	0.794	0.007
pgs000579	122/122	0.602	0.808	0.779	-0.029	0.787	0.789	0.002
pgs000580	118/118	0.616	0.808	0.784	-0.023	0.787	0.794	0.007
pgs000581	377/377	0.627	0.808	0.775	-0.033	0.787	0.789	0.002
pgs000582	377/377	0.627	0.808	0.775	-0.033	0.787	0.789	0.002
pgs000583	1119311/1119311	0.651	0.808	0.771	-0.037	0.787	0.788	0.001
pgs000584	1120596/1120596	0.648	0.808	0.770	-0.038	0.787	0.788	0.001
pgs000585	1300/1301	0.648	0.808	0.777	-0.031	0.787	0.792	0.005

PRS	SNPs matched / SNPs in PRS	PRS Only	Total PSA	PRS + Total PSA	Change in AUC from Total PSA Alone	4Kscore	PRS + 4Kscore	Change in AUC from 4Kscore Alone
pgs000586	1023/1023	0.649	0.808	0.779	-0.029	0.787	0.795	0.008
pgs000587	26400/26418	0.656	0.808	0.777	-0.030	0.787	0.794	0.007
pgs000588	178191/178259	0.652	0.808	0.775	-0.032	0.787	0.792	0.005
pgs000589	42/42	0.591	0.808	0.774	-0.033	0.787	0.783	-0.004
pgs000590	1119236/1119236	0.599	0.808	0.781	-0.027	0.787	0.785	-0.002
pgs000591	79/80	0.608	0.808	0.773	-0.034	0.787	0.783	-0.005
pgs000592	1323/1334	0.589	0.808	0.779	-0.029	0.787	0.788	0.001
pgs000662	269/269	0.649	0.808	0.778	-0.030	0.787	0.795	0.008
pgs000714	55/55	0.526	0.808	0.797	-0.010	0.787	0.779	-0.008
pgs000719	134/134	0.625	0.808	0.783	-0.024	0.787	0.797	0.009
pgs000733	49/49	0.564	0.808	0.778	-0.029	0.787	0.786	-0.001
